# Tourism Experience and Construction of Personalized Smart Tourism Program Under Tourist Psychology

**DOI:** 10.3389/fpsyg.2021.691183

**Published:** 2021-07-22

**Authors:** Feiya Lan, Qijun Huang, Lijin Zeng, Xiuming Guan, Dan Xing, Ziyan Cheng

**Affiliations:** ^1^Faculty of International Tourism and Management, City University of Macau, Macao, China; ^2^Faculty of Law, Hebei University, Baoding, China; ^3^School of Public Economics and Administration, Shanghai University of Finance and Economics, Shanghai, China; ^4^School of Business, Macau University of Science and Technology, Macao, China; ^5^Department of Environmental Art and Design, China Academy of Art, Hangzhou, China

**Keywords:** personalization, smart tourism, Macau tourism, tourism psychology, tourism experience

## Abstract

The present work aims to boost tourism development in China, grasp the psychology of tourists at any time, and provide personalized tourist services. The research object is the tourism industry in Macau. In particular, tourists' experiences are comprehensively analyzed in terms of dining, living, traveling, sightseeing, shopping, and entertaining as per their psychological changes using approaches including big data analysis, literature analysis, and field investigation. In this case, a model of tourism experience formation path is summarized, and a smart travel solution is proposed based on psychological experience. In the end, specific and feasible suggestions are put forward for the Macau tourism industry. Results demonstrate that the psychology-based smart travel solution exerts a significant impact on tourists' tourism experience. Specifically, the weight of secular tourism experience is 0.523, the weight of aesthetic tourism experience is 0.356, and the weight of stimulating tourism experience is 0.121. Tourists prefer travel destinations with excellent urban security and scenic authenticity. They give the two indexes comprehensive scores of 75.14 points and 73.12 points, respectively. The proposed smart travel solution can grasp the psychology of tourists and enhance their tourism experiences. It has strong practical and guiding significances, which can promote constructing smart travel services in Macau and enhancing tourism experiences.

## Background

While China's national strength is improving continuously, people's requirements for the quality of life also becomes higher, and tourism expenditure has increased its share of all living consumption expenditures (Sun et al., 2020). Tourism is an important indicator to measure the happiness and life satisfaction of people; it also reflects the level of living standards. In China, the central government proposed a strategic plan for the development of smart tourism in 2011 (Watson et al., 2017). Supported by the national tourism policy, many smart tourism cities have emerged one after another. The key to smart tourism is to integrate tourism data, including traffic, weather, management, passenger flow, and other data that need to be integrated and considered (Gretzel et al., 2015). Before tourists leave for their destinations, various types of consultation, navigation, and information-sharing services are very critical. As the internet advances, applying new information technology to the tourism industry has become a general trend; as a result, data of the tourism industry has become a hot issue (Alaei et al., 2019). Big data technology is developed on the basis of information technologies, including the internet and cloud computing. This technology plays a vital role in developing tourism products, improving tourism services, and tourism marketing (Lv et al., 2019). A smart tourism service platform is built according to the data of the tourism industry to provide tourists with diversified services and make the tourism experience more personalized and authentic. This model is of great significance for promoting the transformation, upgrading, and sustainable development of the tourism industry.

The smart tourism is centered on the personalization of tourists. Supported by the new generation of communication and internet technologies, smart tourism increases interactive experience, gathers tourism information, and promotes the upgrading and transformation of the tourism industry (Skavronskaya et al., 2020a). The research on psychology-based tourism experience focuses on the travel psychology and preferences of tourists. Analyzing several reports on tourism psychology, Cicerali et al. (2017) found that the most critical factors that harmed tourism satisfaction among tourism environmental factors were sanitary conditions, social influence, scenic area design, and tourism atmosphere. Studying the negative psychology of tourists, Nawijn and Biran (2019) discovered that different types of negative emotions would affect the lives of consumers, while traveling could promote the emotional experiences and improve the negative emotions. Skavronskaya et al. (2020b) proposed a conceptual model called “cognitive evaluation of novelty in unforgettable tourism experience.” They believed that future works should consider applying this model to advance the tourism experience and analyze such experience as a psychological phenomenon. However, the existing methods cannot solve the problems hindering the sustainable development of tourism fundamentally. Therefore, constructing a smart tourism platform based on tourists' psychology is a critical and urgent issue in the tourism industry.

Therefore, influencing factors of the smart tourism industry are analyzed to clarify the specific evaluation indexes. Besides, three tourism experiences, namely secular tourism experience, aesthetic tourism experience, and stimulating tourism experience, are analyzed from six perspectives: dining, living, traveling, sightseeing, shopping, and entertaining. At last, a personalized smart tourism platform founded on tourism psychology is proposed. Through simulation experiments, the platform's effectiveness is validated; on this basis, countermeasures and suggestions are put forward for constructing the smart tourism of Macau. To sum up, a smart tourism platform is built using data mining technology, which can promote the smart tourism development in Macau and provide a basis for the sustainable development of Macau's tourism industry.

## Literature Review

### Related Works of Smart Travel

Smart travel uses new technologies such as cloud computing and the Internet of Things (IoT) to actively perceive information about tourism resources, tourism economy, tourism activities, and tourists through the internet or mobile internet using portable terminal internet devices. It then timely releases the perceived information, allowing people to access the information they need in time to arrange their schedules. Eventually, intelligent perception and convenient use of all kinds of travel information can be achieved (Kharisma and Muni, 2017). Smart travel can be reflected in tourism management, tourism services, and tourism marketing. When people propose the concept of smart travel, they put forward various thoughts on smart travel as per different research directions (Cui and Long, 2019). Li et al. (2017) believed that smart travel was a unique creative tourism. Liberato et al. (2018) thought that smart travel was first a change in the concept of development. Buhalis (2019) pointed out that smart travel was an integration of the new generation of information and communication technologies. Femenia-Serra and Neuhofer (2018) researched the development momentum of smart travel regarding its driving factors. Thakuriah et al. (2020) introduced the relationship between smart city and smart travel and expounded the role of smart travel from multiple angles. Shafiee et al. (2019) introduced the history, framework, value, and development trend of smart travel. Gretzel and de Mendonça (2019) explained the deficiencies of smart travel. Sun et al. (2019) researched smart travel according to the current situation and problems, development countermeasures, and development prospects. Gretzel and Koo (2021) proposed to build and manage a “smart travel public service platform.” Smart travel is a significant innovation in the tourism industry. Its innovation path is formed based on the efficient flow and effective integration of tourism information in the tourism industry. The innovative means include the internet, big data, cloud computing, and other new-generation information technologies, as well as business model innovation. Ultimately, the purpose of innovation is to improve tourism services and tourists' satisfaction.

### Development and Application of Big Data

Big data refers to a collection of data whose content cannot be captured, managed, and processed with conventional software tools within a time frame. Big data technology can quickly obtain valuable information from various types of data. Technologies applicable to big data include massively parallel processing databases, data mining grids, distributed file systems, distributed databases, cloud computing platforms, the internet, and scalable storage systems (Le et al., 2019). Lately, new technologies such as IoT, artificial intelligence, and cloud computing have been accepted in various fields. Chen et al. (2019) believed that these new things were inseparable from the support of big data. A large number of research results have also emerged in the process of assisting in the transformation and upgrading of various industries (Chen et al., 2019). Zhu et al. (2019) proved that combining big data and cloud computing could give new value to the data held by operators. Liu et al. (2020) suggested that big data could bring new ideas to the operation and management of the hotel industry. Du et al. (2020) believed that big data would contribute to tourism management and the development of global tourism. As mobile internet and big data develop rapidly, research on smart travel has gone beyond the theoretical level; scholars begin to combine smart travel with big data and cloud computing to explore a way to practice smart travel (Du et al., 2020). Joubert et al. (2021) studied the operation mode of smart travel by combining value chain management, supply chain management, and other operation management theories. Gao (2021) improved the practicability of smart travel by studying the technical implementation methods behind smart travel. They also explored the combination of smart travel and rural tourism from different angles.

### Related Works of Tourism Psychology

People participating in tourism activities include actual tourists, potential tourists, and various practitioners of the tourism industry. They have different psychological activities in tourism activities and therefore behave differently (Skavronskaya et al., 2020c). There are always contacts and connections among tourists, “tourism products,” tourism service personnel, and tourism enterprise management personnel in tourism activities. These mutual contacts and interpersonal relationships depend on people's psychological activities. Tourism psychology studies the laws of these people's psychological activities and behaviors in tourism activities. Psychological activities and behaviors are inseparable. Psychology governs behavior, and behavior reflects psychology (Kesenheimer and Greitemeyer, 2021). Tourism experience is a comprehensive experience based on super-utilitarian experience. While enjoying this experience, tourists can obtain aesthetic pleasure by observing the scenery, appreciate a colorful life in the interaction with others, discover and develop themselves in the process of actively imitating other roles, and also relish secular pleasures through tourism consumption.

### A Review of Related Works

Related works analyzed above suggest that research results of big data, smart travel, and platform operation are very rich after decades of exploration. These findings provide important ideas and methods for designing smart travel platforms and operating systems based on big data, laying a firm theoretical foundation. However, previous works rarely discuss how to give full play to the important role of big data in global tourism, how to build a new model of smart travel platform operation, how to promote the development of the modern tourism service industry, and how to adapt to the upgrade of tourism consumption needs (Elizabeth et al., 2021). Smart travel is not just the internetization of the traditional tourism industry that is common in the current “Internet +” era, such as “Internet + travel e-government,” “Internet + travel e-commerce,” and “smart scenic spots.” New issues often appear during development, which must be solved through new technologies.

Regarding new demands and new problems, the deep integration of modern big data technology and traditional tourism has created a new operation model for tourism platforms, called smart travel. Because of the differences in tourism informatization and smart travel research worldwide, domestic and foreign tourists have big differences in tourism behaviors; in particular, domestic tourists pay more attention to sightseeing, while foreign tourists pay more attention to leisure. Therefore, there are different tendencies toward smart travel research. For example, the research on smart travel in foreign academia is biased toward the tourism informatization. In contrast, coincided with the explosion of innovation in China due to the demographic dividend, the domestic academia focuses on defining smart travel from different aspects, such as the theory of management changes derived from the research on how information technology affect the management of tourism enterprises, the theory of technology application derived from directly applying information technology to the tourism industry, and the theory of how information technology affects the tourism experience from the perspective of tourists. However, most of these works focus on researching the concepts of smart travel; the essence of smart travel is rarely discussed, and the research on the relationship between big data and smart travel and studies taking smart travel as the core are seldom reported, which can hardly reference the actual smart travel practice.

## Methodology

### SWOT Analysis of Tourism Industry in Macau

SWOT analysis discusses the strengths, weaknesses, opportunities, and threats of the research object to formulate policies accordingly (Peng, 2019). As the leading industry in Macau, tourism plays a significant role in coordinating and consolidating Macau's economic development. Through the SWOT analysis, the advantages and opportunities of Macau's tourism industry can be utilized to make improvements; meanwhile, the disadvantages and deficiencies encountered in the developmental process can be adjusted and upgraded.

#### Strengths Analysis

(1) Macau is located between Hong Kong and Guangdong Province, China. During its development, Macau can take advantages of Hong Kong's convenient seaport transportation and international background, as well as Guangdong's rich human resources and vast market. Macau has a vast potential market, and simple entry procedures have attracted tourists from all over the world. (2) Macau, as a platform for cultural exchanges between China and the West, continues to develop more broadly under the background of inheriting Chinese traditional culture and integrating Western culture. Macau's unique advantage has played an essential role in opening up the mainland and foreign markets, especially in cooperation with Portuguese-speaking countries. (3) Although Macau has a small land area, it has many natural and cultural resources. The historic city of Macau, which has a long history, has been listed as a United Nations cultural heritage. Tourists to Macau can feel the local customs and appreciate the long history and culture of Macau. On the one hand, these cultural resources have greatly enhanced tourists' yearning for Macau. On the other hand, the development of the tourism industry has also promoted the upgrading of other industries to better serve tourists.

#### Weaknesses Analysis

(1) Because Macau has a small land area, a large population, and not too many important enterprises, tourism has always been a pillar industry of Macau. This is undoubtedly a significant drawback for a city seeking comprehensive development. Adjusting the industrial structure and realizing all-round industrial development is the direction of Macau's continuous advancement of reform. (2) Macau is close to Hong Kong. Many tourists drop by Macau after visiting Hong Kong. Macau's convenient transportation also makes many tourists choose to go to Hong Kong or Zhuhai, Guangdong instead of staying in Macau after a day of sightseeing. According to statistics, the average time of tourists staying in Macau is 1.4 days, which is much lower than the time that tourists stay in the true sense. Therefore, Macau needs to speed up the construction of supporting facilities, add a wealth of tourism projects, and attract the attention of tourists as much as possible to extend the stay time of tourists. (3) The rapid development of tourism is inseparable from the support of human resources. As the training of talents cannot keep up with the development of the tourism industry, gaps in professional talents and job vacancies appear, which limits the development of Macau's tourism industry to some extent.

#### Opportunities Analysis

(1) The support of national policies and the influence of surrounding areas have brought new development opportunities to Macau's tourism industry. Macau is backed by mainland China and facing foreign markets; it is supported with a strong human market and resources. The advantages of “One Country, Two Systems,” the construction of the Guangdong-Hong Kong-Macau Greater Bay Area, the establishment of the Guangdong Free Trade Zone, the signing of the *Guangdong-Macau Cooperation Framework Agreement*, and the implementation of “The Belt and Road” initiative all contribute to the development of Macau's tourism industry and the overall economy, infusing the city with vitality and vitality. (2) Macau can exploit various types of tourism and develop the exhibition industry. Macau has the intersection of Chinese and Western cultures, which significantly promotes the “going out” and “bringing in” of the local economy. Enterprises in mainland China hope to strengthen cooperation with international enterprises via Macau, and international enterprises can enter the vast mainland market via Macau. The development of the convention and exhibition industry not only promotes the development of tourism but also increases the visibility of tourist destinations, attracts more tourists, and extends their stay time. Moreover, it has also promoted local economic development. The improvement of various large-scale infrastructures has attracted more investment and strengthened scientific and technological exchanges with different countries and regions (Liu and Li, 2019; Li, 2019).

#### Threats Analysis

(1) The narrow land area and inconvenient transportation restrict the further development of tourism. The imbalance between Macau's land area and population makes the tourism infrastructure incomplete. Famous scenic spots, such as Ruínas da Antiga Catedral de São Paulo, Largo do Senado, and Avenida de Almeida Ribeiro, will be overcrowded during the holidays, which will not only affect road traffic but also induce safety accidents. (2) The competition is fierce in the surrounding tourism market. As tourists continue to travel abroad, more countries and regions begin to focus on developing tourism. The rapid development of tourism in Southeast Asia has impacted Macau's tourism. Fierce market competition makes Macau tourism industry have to face a new round of reforms and upgrades (Su and Zhao, 2019).

The above analysis reveals the following demands: (1) the demand for tourism industry development. The current development of Macau's tourism industry has entered a tough period of transformation and upgrading. The imbalance between the supply and demand structure of the tourism market is very prominent. The development and operation modes of the industry are relatively traditional. (2) The demand for liberalized, diversified, and personalized tourism consumption. Looking up information and booking travel services anytime and anywhere are new demands for tourism consumption. This has put forward an unprecedented high standard of demand for the comprehensiveness, vividness, and detail of tourism public information services. (3) The demand for the transformation of service-oriented government functions and improvement of administrative efficiency. In the past, Macau's tourism industry was supervised and supported by government administration. They have acted as rule-makers and executors more often. With the deepening of marketization and the development of modern information technology, this type of management cannot solve tourism problems in Macau during the development of the industry.

### Smart Travel Big Data Analysis Platform Based on Resources and Psychology

**Figure 1:**
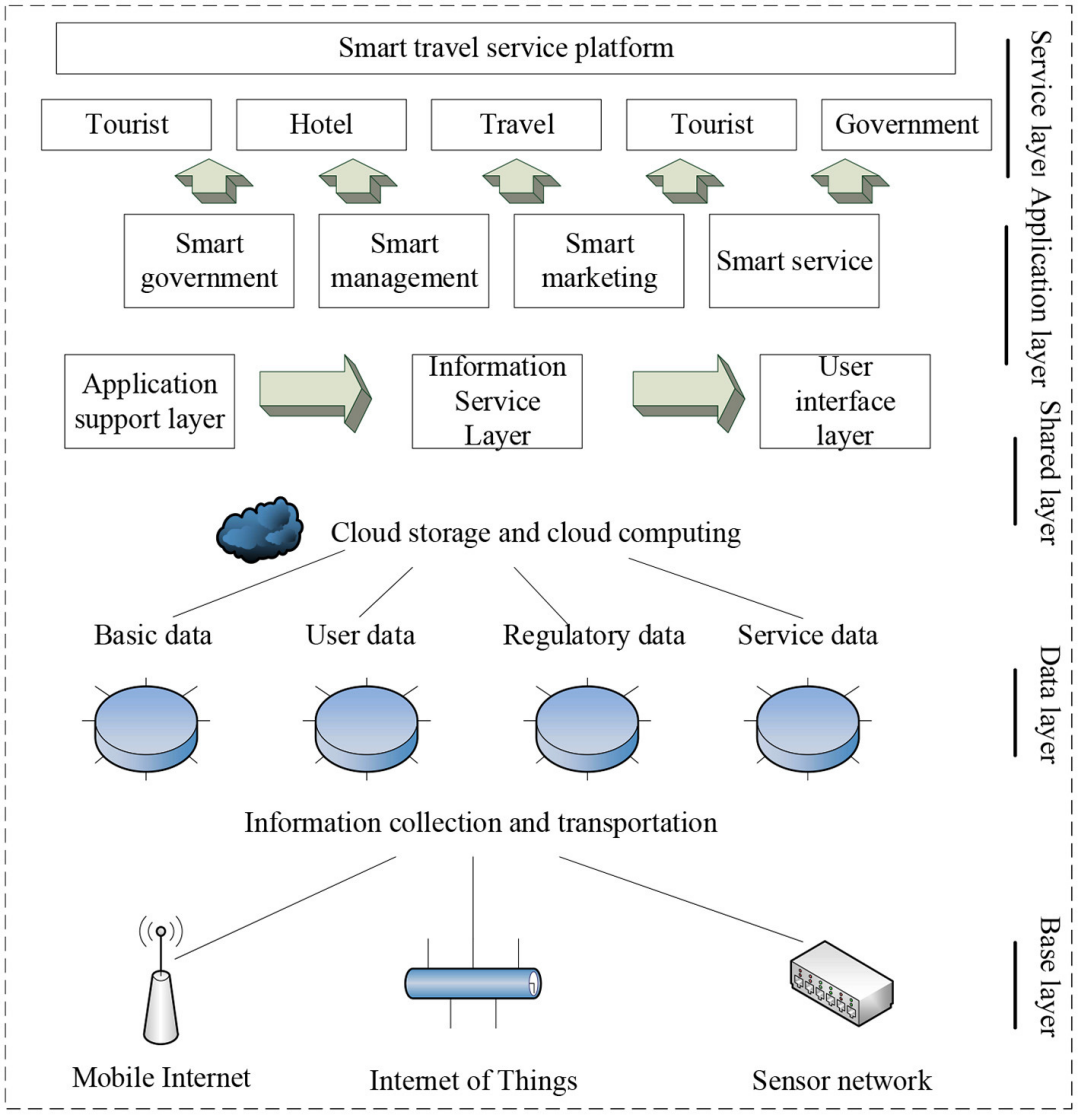
The standard system of smart travel service platform.

The present work is based on the resource-based smart travel service platform. On this basis, big data processing methods are added to analyze the impact of the smart service platform through different indexes. Hence, a big data travel service platform that is more suitable to the Macau region can be proposed through data optimization. The analysis and application of big data can ensure the sustainable development of tourism (Ardito et al., 2019). Figure 1 presents the structure of the designed platform.

**Figure 2:**
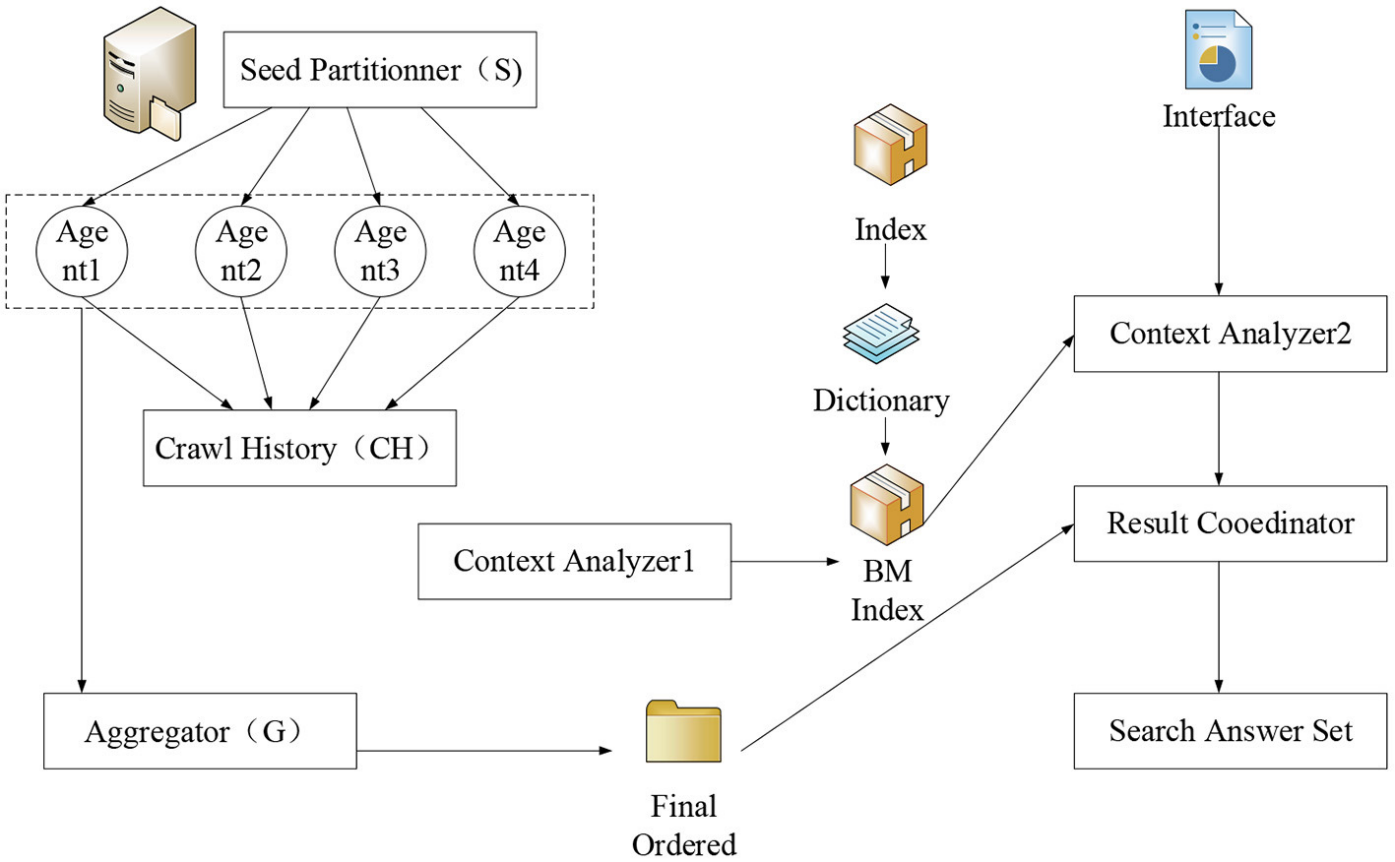
Schematic diagram of web crawler data scraping steps.

Data used in the present work are collected through data mining, including data collection, data analysis, and result analysis. Figure 2 shows the data collection method of web crawlers. Data can also be obtained through third-party purchase (after filtering out the privacy information, data about the user's consumption status and ability are obtained). The tourism data can be captured in real-time through the above approaches. Government and enterprises need to fill in and report the data. Hence, the personnel need to enter the big data platform and input the information manually to ensure the data integrity. The collected data are analyzed and compared using algorithms according to the specific knowledge base and the corresponding database to draw and visualize the conclusions (Del Vecchio et al., 2018). Data mining is applied at the most basic layer of the model. Different classifications and algorithms are practiced to achieve the best model efficiency and ensure the integrity of the information on the travel service platform.

**Figure 3:**
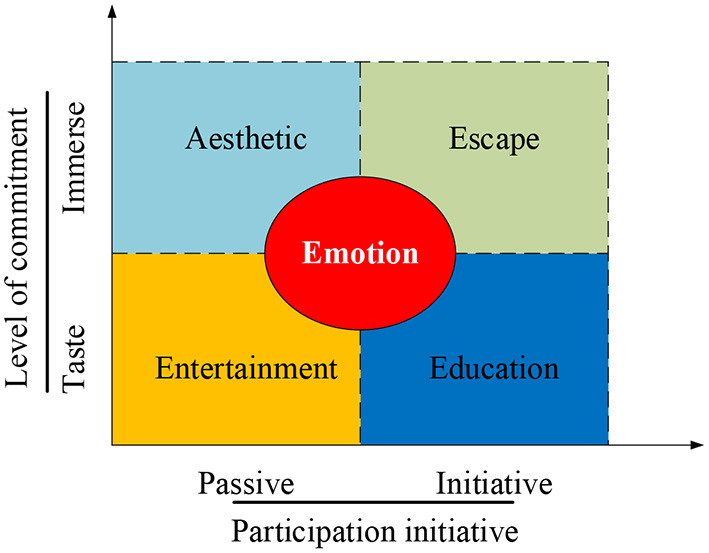
“4E” model based on tourism experience.

Tourism experience refers to the visual aesthetic experiences and the spiritual experience that tourists feel during traveling, such as learning and cognition; tourists not only observe the external expressions of things but also think about the rational world (Sedera et al., 2017). As one of the representative studies, Luo et al. (2018) classified tourism experience in their research. They believed that the ultimate pleasure of tourists through compensation or realization was defined as travel pleasure. Pleasure was the core of the tourism experience, and the purpose of the tourism experience was to seek happiness or pleasure. Pleasure could be divided into tourism aesthetic pleasure; that is, the pleasure obtained through transcendental tourism experience was tourism secular pleasure, which was the pleasure obtained through regressive tourism experience (Luo et al., 2018). The so-called secular tourism pleasure is the usual pleasure form of entering life. It is based on the utilitarian understanding of the perceived object through other organs other than the audiovisual senses. It is the collective term for all the pleasures in addition to the aesthetic pleasure experienced by tourists during the traveling process. The prerequisite and intensity of secular pleasure are related to the accumulation of previous experience, which varies with time, place, person, and event. It is a kind of pleasure that is obtained by a single low-level sense organ (such as touch, taste, and smell) other than audiovisual. The aesthetic tourism pleasure is the primary goal of tourism experience. It is a kind of psychological experience that gets rid of the sense of interest and utilitarianism. It refers to a psychological experience generated by tourists when they appreciate the beautiful nature, artwork, and other artificial products. In essence, experience is “a comprehensive aesthetic practice that integrates natural beauty, artistic beauty, and the beauty of social life.” Figure 3 shows the “4E” tourism experience model proposed by Pine and Gilmore (Santos et al., 2019). According to Pine and Gilmore, the essence of tourism is to obtain a pleasant experience. They divide the pleasure of tourism into aesthetic pleasure and secular pleasure.

### Indicator Analysis and Model Construction

Literature about tourism psychology is reviewed and reorganized to build the three-level index system. As shown in Table 1, the system includes the secular experience, the aesthetic experience, and the stimulating experience (Li, 2019; Li et al., 2020). The secular experience is reflected in dining, living, and sightseeing, including food safety, food delicacy, accommodation safety, accommodation comfort, and traveling convenience. The aesthetic experience is reflected in the sightseeing activities, including the convenience of sightseeing, the condition of nature/culture, and the comfort and safety of sightseeing trips. The stimulating experience is reflected in the freshness and stimulus brought by shopping and entertaining.

**Table 1:** Construction of smart tourism index system based on tourist psychology.

**Target layer**	**Criterion layer**	**Object layer**	**Secondary indexes**
Overall decision level	B1-Secular experience	P11-Food hygiene	Food testing compliance, health, and safety.
		P12-Food delicacy	Delicious, cheap, distinctive, and flavorful.
		P13-Accommodation security	Own financial security, personal safety, and hotel staff mobility.
		P14-Accommodation comfort	Hotel health, services, supporting facilities, and follow-up.
		P15-Travel convenience	Convenient, fast, comfortable, and cheap.
	B2-Aesthetic experience	P21-Convenience	Convenient, fast, the number of tourists in scenic spots, traffic convenience, and traffic conditions.
		P22-Natural/cultural restoration	The authenticity of the original features of natural scenic spots and the reduction of cultural scenic spots.
		P23-Play safety	Whether the safety measures for play are sufficient and whether the signs of dangerous areas are in place.
		P24-Play comfort	Whether the scenic area can bring pleasant feelings and happiness.
		P31-Freshness	Data indexes, data Arouse people's inquiry and curiosity about new things.
	B3-Stimulate the experience	P32-Irritation	Give people an unprecedented sense of stimulation and increase pleasure.

**Figure 4:**
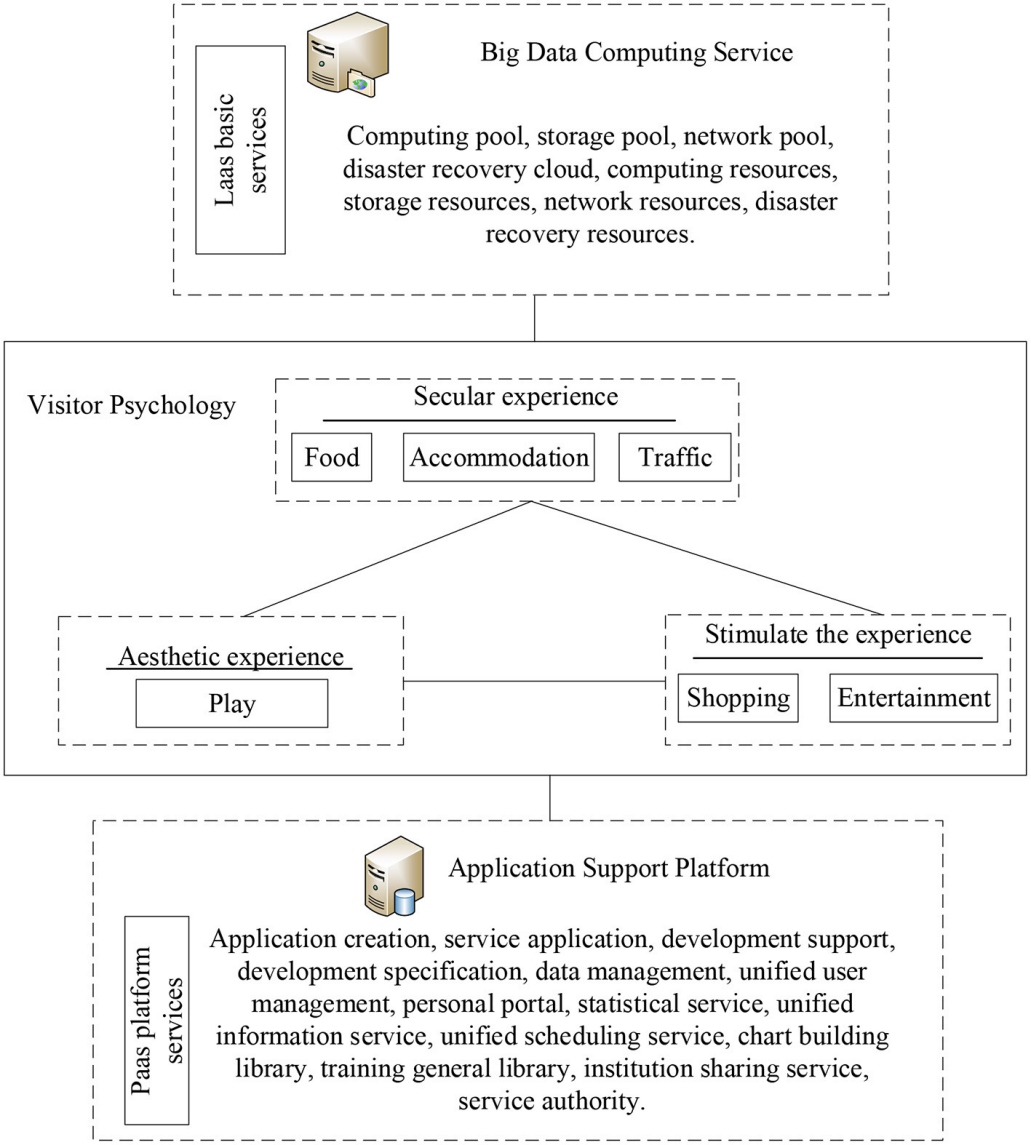
The smart tourism platform based on tourist psychology.

According to the investigations of literature, models, and data, problems in the smart tourism construction in Macau are analyzed. On this basis, a smart tourism platform based on tourism psychology is built, as shown in Figure 4. This platform specifically includes: (1) the basic service layer: this layer adopts the big data processing method. It includes the functions of data analysis and data collection, such as the corresponding calculation rule, storage pool, and network pool. (2) The psychological analysis of tourists: the tourism experience is divided into secular experience, aesthetic experience, and stimulating experience according to the consumption data and consumption-ability of tourists. These three experiences are analyzed from the six perspectives: dining, living, traveling, sightseeing, shopping, and entertaining to draw the psychological prediction of tourists. (3) The software service layer: as per the predicted psychological data of tourists, the software service layer is oriented to the special application subsystem, which implements business applications such as real-time passenger flow analysis and prediction, tourist value prediction, passenger flow monitoring analysis, and satisfaction index analysis. At the same time, the software can be expanded and updated. The system not only runs independently but also exchanges and shares data, which continuously expands the functions of the smart tourism service platform.

### Model Performance Evaluation and Data Sources

**Figure 5:**
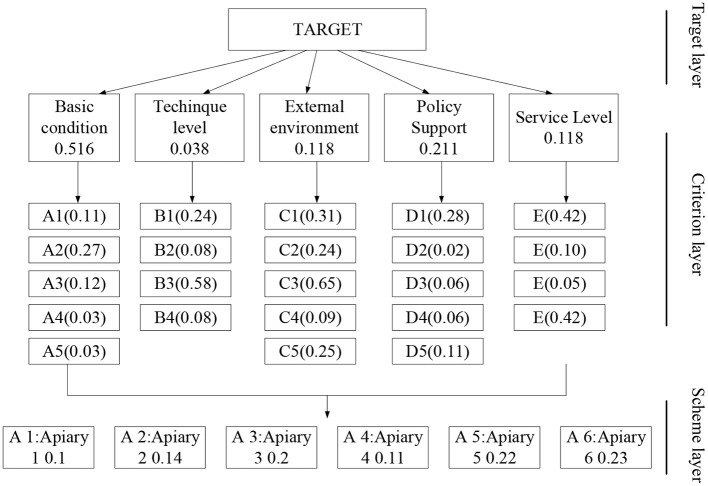
The AHP structure of Macau's tourism industry.

Analytic hierarchy process (AHP) is a qualitative and quantitative analysis tool that classifies factors affecting the decision-making according to the target layer, the criterion layer, and the plan layer. Through AHP, the optimal solution can be obtained (Ho and Ma, 2018). Figure 5 illustrates the AHP structure of Macau's tourism industry. Here, factors affecting the services of Macau's tourism industry are determined by the scaling method. Besides, the opinions of experts are combined to score the indexes objectively. Suppose that *W*_*n*_ represents the variable of the matrix, and aij refers to a collection of various variables. In that case, the judgment matrix between them is:

(1) $$\begin{array}{l} \text{A}=\left(\begin{array}{l} \begin{array}{l} \begin{array}{l} W_{1}/W_{1}\cdots \;W_{1}/W_{\text{n}}\\ \vdots \end{array}\\ W_{\text{n}}/W_{1}\cdots W_{\text{n}}/W_{\text{n}} \end{array} \end{array}\right)\\ \;={\left(\text{aij}\right)}_{\text{n}}\times \text{n} \end{array}$$

In the meantime, (1) (2) a_*i*j_ = 1aji, (*I*, j = 1, 2, 3, ⋯*n*) (3). Therefore, the following equation is obtained:

(2) $$\begin{array}{l} \text{AW}=\;\left(\begin{array}{l} W_{1}/W_{1}\cdots W_{1}/W_{\text{n}}\\ \begin{array}{l} \;\vdots \\ \;W_{\text{n}}/W_{1}\cdots W_{\text{n}}/W_{\text{n}} \end{array} \end{array}\right)\\ \;=\left[\begin{array}{l} W_{1}\\ \;W_{2}\\ \;...\\ \;W_{n} \end{array}\right]=n\;\left[\begin{array}{l} W_{1}\\ \;W_{2}\\ \;...\\ \;W_{n} \end{array}\right]=\text{nW} \end{array}$$

The experimental environment is summarized in Table 2 below. The operating system is Microsoft Windows 10. The platform is written in Python. The Oracle10g database is used as the basis for building the network framework.

**Table 2:** Experimental development environment.

**Resource type**		**Model**
Development PC	Model	ASUS X555YI
	Operating system	Windows 10
	Processor	Inter (R) Xeon (R) CPU E5-2650 v4
	RAM	128G
	System type	64-bit operating system
	Graphics card	Tesla M40
Software configuration	Dependency library	Oracle10g
	Language	Python
Development engine	Version	2017.2.0f3
	Script editor	Visual Studio 2017
	Compiled language	C/C#
	AR SDK	Vuforia

Questionnaires are distributed to survey whether this platform is helpful to the tourism experience. The questionnaire is designed according to the indexes in the performance evaluation. For each question, there are five options: “Excellent,” “Good,” “Fair,” “Poor,” and “Very Poor,” corresponding to 1, 0.8, 0.6, 0.4, and 0.2 points, respectively. A total of 300 questionnaires were issued, and 285 were returned, of which 270 were valid. The response rate is 95%, and the valid rate is 94.7%. The statistical software is utilized to analyze the reliability and validity of all questionnaires for subsequent in-depth research and analysis.

There are three data sources: (1) data provided on the Macau official tourism website are collected. The Macau tourism department is interviewed through telephone to obtain first-hand field survey data. Through analysis, summary, and induction, the problems in the development of Macau's tourism industry are summarized. (2) Comparative analysis: smart travel platforms in different provinces are compared to find feasible methods for the Macau tourism industry. In practical applications, the shortcomings and deficiencies of traditional tourism in management, marketing, and services are listed and compared with big data processing results to show the role of big data in the practical application of tourism. (3) Interview: heads of the Macau Tourism Bureau and Macau Regional Tourism Bureau are interviewed to understand the problems of Macau smart travel. Moreover, heads of related technology enterprises such as Beijing Golden Bridge Network Communication Co., Ltd. are interviewed.

The questionnaire survey is conducted to verify and improve the research design of smart travel ways and improve the tourism experience. The purpose is to verify whether the smart travel methods currently applied are helpful to the improvement of the tourism experience. The Forbidden City is added to the questionnaire as a case site. Through specific cases, it is hoped to understand the impact of smart travel on the quality of tourism experience in the current application. According to Sthapit's tourism experience model (Sthapit et al., 2019), the influencing factors in the interference variables include delay, comfort, convenience, accessibility to the destination, the nature of the destination, the quality of accommodation, the number of attractions and activities, and the ethnic nature of the destination. The influencing factors in the interaction process include the gap between actual feelings and expectations, the nature of the interaction with the destination residents and fellow tourists, the ability to distinguish the authenticity and illusion of events, the ability of psychological adjustment, and the ability to communicate. According to the nature of the case, the four factors of comfort, convenience, the gap between actual feelings and expectations, psychological adjustment, as well as the satisfaction of tourists' overall tourism experience, are selected. The questionnaire is designed according to the indexes in the performance evaluation; each question has 5 options: “Excellent, Good, Fair, Poor, and Very Poor,” corresponding to 1, 0.8, 0.6, 0.4, and 0.2 points, respectively. Based on the indicator weights of the smart tourism platform of tourism psychology are shown in Table 3 above. A total of 300 questionnaires were sent out, and 285 were returned. Among them, 270 were valid questionnaires, with a response rate of 95% and a valid rate of 94.7%. Statistical software is employed to analyze the credibility and validity of all questionnaires.

**Table 3:** Index weight of the smart tourism platform based on tourism psychology.

**Smart tourism platform based on tourist psychology**	**B1-Secular experience**	**B2-Aesthetic experience**	**B3-Stimulate the experience**	**Weights**
B1-Secular experience	1	1.221	0.379	0.356
B2-Aesthetic experience	3.187	1	3.215	0.523
B3-Stimulate the experience	0.732	0.245	1	0.121

*In the table, λmax = 3.030; CI = 0.001; RI = 0.58; CR = 0.001; CR < 0.1; the consistency has been verified*.

## Results

### Effectiveness of the Smart Tourism Platform

**Figure 6:**
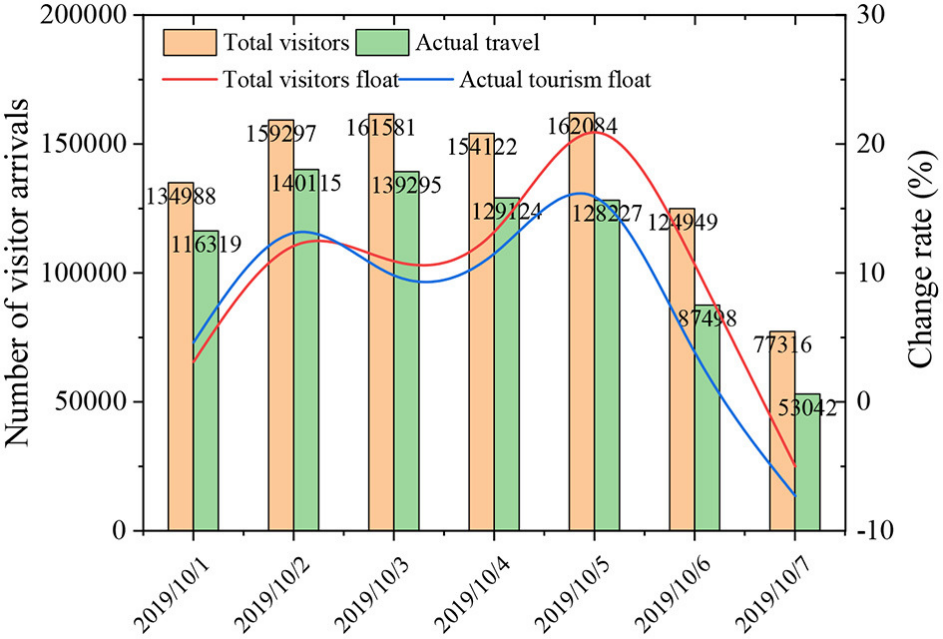
Results of statistical analysis of Macau's tourism data during the National Day Holidays in 2019.

The visual data of the smart travel platform based on tourism psychology are illustrated in Figure 6. In particular, the geographical distribution of tourists in Macau can be collected more accurately through this platform. Through the crawling rules, the system accurately counts the total number of tourists entering Macau. Changes in the number of tourists during 1 week are as follows: the number of tourists begins to increase on Oct. 1st; on Oct. 2nd, it continues to increase; on Oct. 3rd, it starts to decrease; on Oct. 5th, it reaches the peak. The actual number of tourists also show the same trend. Besides, the daily change index is analyzed. The curve can reveal the trend of tourists entering Macau during the National Day Holidays. These results show that the smart tourism platform based on tourism psychology has strong data mining and analysis capabilities, and the visual display effect is noticeable.

### Index Weight of the Smart Tourism Platform

Table 1 shows the result of the index weight analysis of the smart tourism platform based on tourism psychology. A detailed analysis of the questionnaire survey data reveal that tourists have a maximum weight of 0.523 for the aesthetic tourism experience, followed by the secular tourism experience, with a weight of 0.356. This shows that the majority of tourists in Macau undergo aesthetic sightseeing.

**Figure 7:**
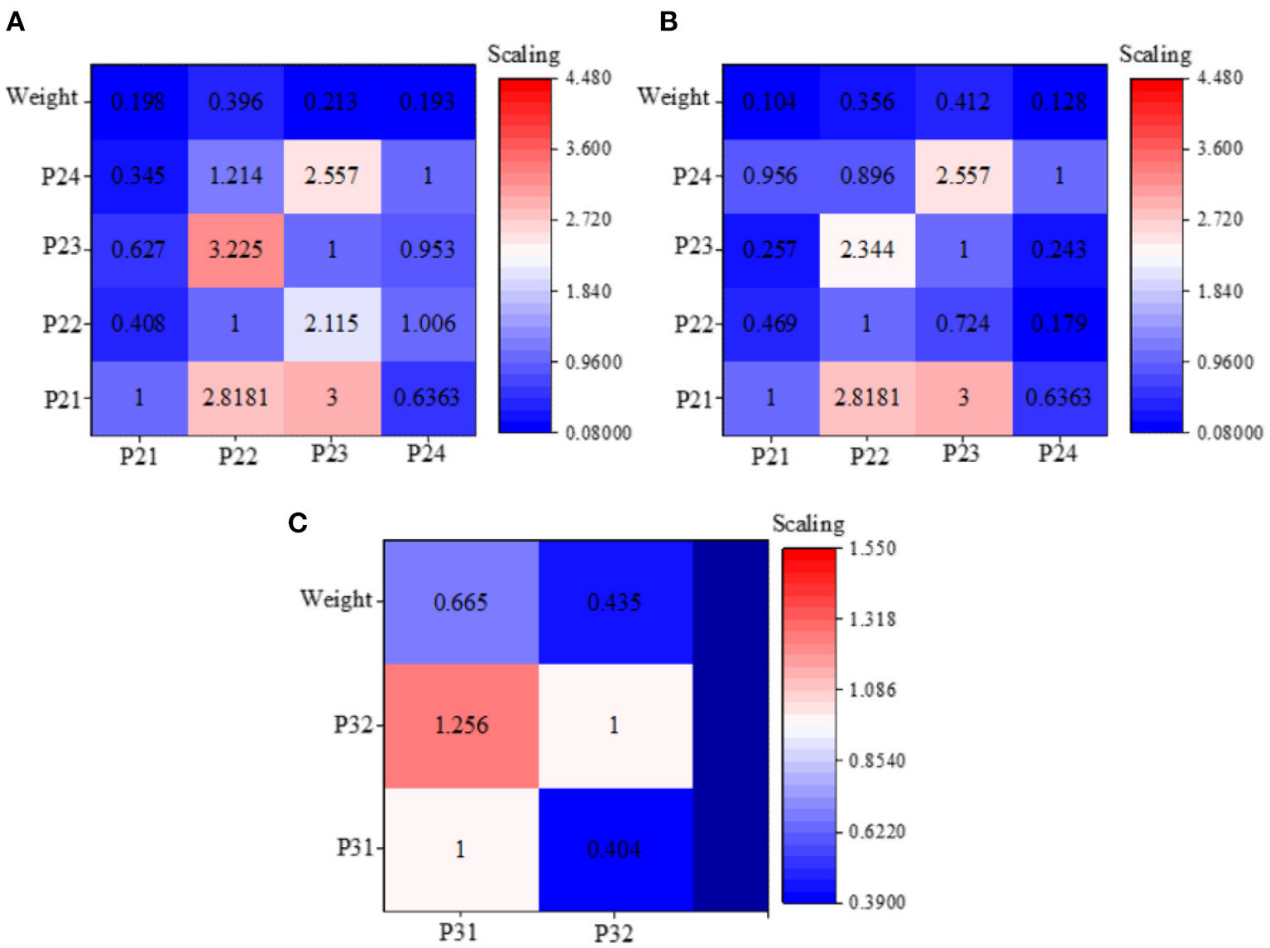
Weighted results of various indexes of the smart tourism platform based on tourist psychology [**(A)** is the technical level index weighting result; **(B)** is the external environment index weighting result; **(C)** is the basic condition index weighting result].

Figure 7 summarizes the results of block analysis on the weights of the platform indexes under different index systems. As shown in Figure 7A, in the secular tourism experience, the largest weight is the taste of food, reaching 0.396. The reason is that most tourists pay great attention to the cuisine of a region, and the taste of food can represent the culture and custom of a region. The weight of accommodation safety ranks second position, reaching 0.198. The aesthetic tourism experience is shown in Figure 7B, where the largest weight is the safety of the scenic area, reaching 0.312. The reason is that the safety of the accommodation determines the degree of pleasure of traveling; traveling is a matter of spending money to enjoy happiness, and such enjoyment will be greatly reduced if tourists spend more money due to personal safety. The stimulating tourism experience is shown in Figure 7C, where the weight of freshness sense ranks first, reaching 0.665. The above results suggest that the safety psychology of dining and living in secular tourism experience affects tourism consumption. In terms of sightseeing, more attention is paid to safety, and the sense of freshness id more important to tourists.

All index weights are analyzed comprehensively, and the results are demonstrated in Table 4. The weights of the criterion layer are 0.378, 0.462, and 0.16, respectively. Impacts coming from indexes of the smart tourism platform in descending order are as follows: secular tourism experience > authentic tourism experience > stimulating tourism experience. The above judgment matrix, single hierarchical ranking, and hierarchical total ranking analysis all pass the consistency test; thus, the calculated weights are acceptable. The results obtained from the above data analysis and block weight comparison are consistent.

**Table 4:** Building an index system for the Macau smart travel service platform based on big data.

**Target layer**	**Criterion layer**		**Object layer**	**Index weight**
Overall decision level	B1-Secular experience	0.378	P11-Food hygiene	0.232
			P12-Food delicacy	0.415
			P13-Accommodation security	0.221
			P14-Accommodation comfort	0.132
			P15-Travel convenience	0.234
	B2-Aesthetic experience	0.462	P21-Convenience	0.146
			P22-Natural/cultural restoration	0.296
			P23-Play safety	0.312
			P24-Play comfort	0.216
	B3-Stimulate the experience	0.16	P31-Freshness	0.625
			P32-Irritation	0.375

### Overall Performance of the Smart Tourism Platform

**Figure 8:**
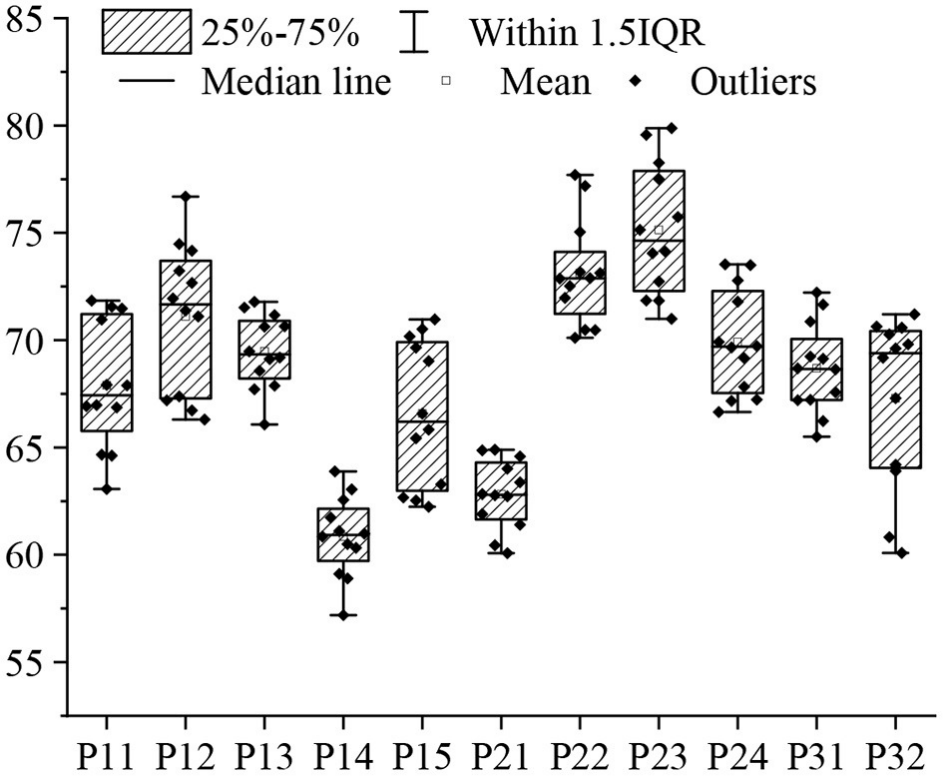
Scoring results of the smart tourism platform based on tourism psychology.

Figure 8 illustrates the scoring results of the designed platform rated by the heads and staff from various industries. This platform receives a comprehensive score of 68.45, which is excellent. Overall, the secular tourism experience accounts for a large proportion because most tourists seek for leisure and entertainment, and therefore their consumptions are normal and average. They prefer destinations and travel plans that can improve mood and pleasure. From a partial perspective, the most important factor that affects the platform is that people consider the total score of tourism safety to be 75.14, followed by the authenticity of the scenic spot, with a comprehensive score of 73.12. This is the most important issue to construct the smart tourism service platform in Macau. The comfort requirement of tourists for accommodation is not very high, which is only 60.85. Therefore, the local tourism department of Macau should reduce its investment in accommodation and increase its investment in the safety and comfort of tourist attractions.

## Discussion and Conclusion

To construct the Macau Smart Travel Service Platform, emphasis should be placed on designing and development according to tourists' travel psychology. This requires a large amount of tourism data. However, in the actual research process, many scenic spots and enterprises cannot realize the importance of data compared to actual benefits, which cannot guarantee the sustainable development of Macau's tourism industry. Therefore, on the basis of the data results, the following suggestions are put forward: (1) the data concepts shall be changed, and the data awareness shall be cultivated, including data openness and sharing concepts, data analysis concepts, and data application concepts. Data involved in tourism works should be emphasized and respected to be used for publicity and services. (2) The major tourism service platforms shall be centralized to collect tourist consumption data. Then, these data shall be analyzed using big data and in-depth mining technologies to find new growth points of the tourism industry from these data. (3) The interaction with tourists shall be strengthened, the communication with tourists shall be deepened through social media such as WeChat and TikTok, and tourists' travel needs shall be understood in time. In this way, a sustainable smart travel service platform can be established, and market-oriented approaches can be applied to mobilize tourism enterprises to raise funds in various aspects and cooperate in constructing part of the smart travel platform.

The government smart travel public service system refers to the general term for public products and services provided by the government or other social organizations that are not profit-oriented, have obvious publicity, and meet the common needs of tourists as the core. It connects tourism suppliers, tourism regulatory agencies, and other tourism market-themed activities in various tourism information demanding links, such as tourism transportation, tourism safety public services, and tourism environmental public services. The public service system of smart travel takes the improvement of tourist satisfaction as the core and the tourism information service as the main body. Its purpose is to meet the needs of individual tourists for the richness, comparability, timeliness, and convenience of obtaining travel information during the dining, living, traveling, sightseeing, shopping, and entertaining process of the travel. For services that can be achieved through market operations, the government should issue relevant policies and implement supportive supervision; moreover, the government should promote the tasks that enterprises are unwilling to do but are related to the overall situation. For example, online services are mainly travel information services provided by local enterprises, aiming to meet the diverse needs of tourists. The government regulates public information services extended by leveraging the nationwide database resources of travel enterprises such as Ctrip.com and eLong.com. The government should lead components of offline smart travel public service infrastructure. The tourism management department also obtains tourist information and real-time market operation data by providing smart travel public services to enhance the timeliness and pertinence of management. In short, by building a smart travel public service system, methods to promote the tourism industry can be changed thoroughly. The level of tourism services can be improved to make tourists travel conveniently. The image of tourism cities can be enhanced, and the supervision of the tourism market can be strengthened to provide tourists with fast, accurate, and comprehensive information services.

From the perspective of tourism experience, smart travel, a new vane in the tourism field, is analyzed. The experience needs of tourists are understood by studying the ways of experience generation. Through the six major elements of tourism: dining, living, traveling, sightseeing, shopping, and entertaining, a research design for smart promotion of tourism experience is constructed, whose usability in practice is then validated, proving that some of the current smart travel measures can improve tourists' tourism experience. This is theoretically innovative. Besides verifying the active role of existing smart travel methods in improving the tourism experience, practical smart travel measures that can improve the tourism experience are proposed, considering the smart travel participants in real-world applications, such as hotels and scenic spots. The smart travel model based on tourist psychology has strong data mining and analysis capabilities, and the visual display effect is obvious. From a psychological perspective, tourists prefer travel destinations with excellent urban security and scenic authenticity. The comprehensive scores for the two are 75.14 points and 73.12 points, respectively. Therefore, Macau's local tourism department should reduce the investment in accommodation and increase the investment in the safety and comfort of tourist attractions.

Despite the constructed big data smart travel platform based on psychology, some weaknesses are found in the present work. First, due to time and research funding issues, only three regions in Macau are surveyed, only covering a small amount of data. Moreover, the questionnaires are mostly distributed on-site. In the future, they can be issued online. Second, because the data of the major tourism service platforms are commercial secrets, only the available network data are analyzed, with a small data amount. Finally, there are few categories of psychology research on tourists, only considering the sense of experience brought by tourism rather than specific consumption data. In the following works, these aspects will be analyzed and research in-depth to realize the practical application of the platform as soon as possible.

## Data Availability Statement

The raw data supporting the conclusions of this article will be made available by the authors, without undue reservation.

## Ethics Statement

The studies involving human participants were reviewed and approved by City University of Macau Ethics Committee. The patients/participants provided their written informed consent to participate in this study. Written informed consent was obtained from the individual(s) for the publication of any potentially identifiable images or data included in this article.

## Author Contributions

All authors listed have made a substantial, direct and intellectual contribution to the work, and approved it for publication.

## Conflict of Interest

The authors declare that the research was conducted in the absence of any commercial or financial relationships that could be construed as a potential conflict of interest.
